# Expanding the genetic and clinical landscapes of hereditary spastic paraplegia (HSP): a cohort study of 103 families

**DOI:** 10.1186/s13023-026-04373-8

**Published:** 2026-05-12

**Authors:** Atefeh Davarzani, Moez Ravanbod, Aida Ghasemi, Mahsa Mohammadi, Mohammad Rohani, Shahriar Nafissi, Payman Jamali, Hossein Najmabadi, Farzad Fatehi, Shahryar Alavi, Zahra Firoozfar, Fariba Zemorshidi, Mohammad Reza Habibi-Kavashkohie, Afagh Alavi

**Affiliations:** 1 Genetics Research Center, University of Social Welfare and Rehabilitation Sciences, Tehran, Iran; 2 Neuromuscular Research Center, Tehran University of Medical Sciences, Tehran, Iran; 3 Department of Neurology, The Five Senses Health Institute, Iran University of Medical Sciences, Tehran, Iran; 4 Neurology Department, Shariati Hospital, Tehran University of Medical Sciences, Tehran, Iran; 5 The Genetic Counseling Center, Shahroud Welfare Organization, Shahroud, Iran; 6 Kariminejad-Najmabadi Pathology & Genetic Center, Tehran, Iran; 7 Visiting Academic, Clinical Neurosciences, Clinical and Experimental Sciences, Faculty of Medicine, University of Southampton, Southampton, UK; 8 Palindrome, Isfahan, Iran; 9 Department of Neurodegenerative Diseases, UCL Queen Square Institute of Neurology, University College London, London, UK; 10 Department of Neuromuscular Diseases, UCL Queen Square Institute of Neurology, University College London, London, UK; 11 Department of Neurology, Mashhad University of Medical Sciences, Mashhad, Iran; 12 CHU Sainte Justine Research Center, University of Montreal, Montréal, Canada

**Keywords:** Hereditary spastic paraplegia (HSP), Whole-exome sequencing (WES), Genetic heterogeneity, SPG11, SPG4

## Abstract

**Background:**

Hereditary spastic paraplegia (HSP) refers to a heterogeneous group of genetic disorders with more than 90 causative genes. Clinically, HSP is classified into pure and complicated forms. Pure forms are characterized primarily by lower-limb spasticity and weakness, whereas complicated forms include additional neurological or non-neurological symptoms alongside spasticity and weakness. We aimed to characterize the clinical and genetic landscapes of HSP in an Iranian cohort. Whole-exome sequencing (WES) was performed on 103 unrelated clinically suspected HSP probands. Multiple ligation-dependent probe amplification (MLPA) was performed to validate identified copy number variants (CNVs) in two probands.

**Results:**

71 pathogenic/likely pathogenic and VUS variants were identified in 81 probands; total genetically solved probands: 78.6%. Among these solved cases, 64 probands harbored variants in known HSP genes, 14 had variants in other neuromuscular/neurodegenerative-related genes, and the remaining 3 probands carried variants in four novel candidate genes including *NMNAT1, SEMA3A, KCNJ14*, and *EMP3*. Among all 71 identified genomic variants, two were CNVs and one was a trinucleotide repeat expansion. Taken together, these variants were located in 37 genes; 21 of these genes have been previously implicated in HSP, and four common HSP subtypes (SPG11, SPG4, SPG7, and SPG15) accounted for ~40% of our cohort.

**Conclusions:**

This study demonstrates significant clinical and genetic heterogeneity of HSP within our cohort. In addition to variants in 21 known HSP-related genes, we identified variants in 14 genes related to other neurological disorders -highlighting shared biological pathways- as well as variants in four novel candidate genes. Notably, a genetic diagnosis could not be established in 22 probands, underscoring that additional, as yet unidentified genes likely contribute to HSP pathogenesis.

**Supplementary information:**

The online version contains supplementary material available at 10.1186/s13023-026-04373-8.

## Background

Hereditary spastic paraplegia (HSP) refers to a heterogeneous group of neurodegenerative disorders. The common and unifying features of HSP include progressive spasticity and weakness of the lower limbs, resulting from axonal degeneration of the upper motor neurons (UMNs) within the corticospinal tract [1]. The prevalence varies by geographic regions, ranging from 0.1 to 9.6 per 100,000 individuals [2].

According to the Harding classification, HSP can be clinically grouped into two main subtypes: pure/uncomplicated/non-syndromic (p-HSP) and complicated/complex/syndromic (c-HSP) forms [3, 4]. Isolated spasticity and weakness of the lower limbs are characteristics of p-HSP, usually accompanied by bladder dysfunction, gait disturbance, hyperreflexia (increased deep tendon reflexes [DTRs]), extensor plantar response (Babinski sign), and sensory symptoms. The last three symptoms typically appear bilaterally in the lower limbs [1, 5]. However, complicated forms present with a combination of neurological and non-neurological manifestations in addition to those observed in p-HSP, including cerebellar ataxia, peripheral neuropathy, epilepsy, parkinsonism, intellectual disability (ID), developmental disorders, short stature, auditory impairment, retinopathy, cataracts, dysarthria, ichthyosis, and respiratory disorder [6, 7].

Genetically, HSP represents one of the most heterogeneous groups of Mendelian disorders. Recent progress in next-generation sequencing (NGS) technologies has revolutionized the diagnosis of HSP-causing genes in the majority of suspected HSP patients [8]. Based on the Online Mendelian Inheritance in Man (OMIM; https://www.omim.org/) database, more than 90 distinct genetic subtypes of HSP (SPG1–SPG93) have been identified to date, each caused by pathogenic variants in a specific gene. These subtypes may be associated with distinct clinical manifestations and inheritance patterns [1]. It may be inherited in an autosomal dominant (AD-HSP), autosomal recessive (AR-HSP), X-linked recessive (XLR), or mitochondrial manner. However, 13–40% of cases can present as sporadic [2, 9, 10]. Additionally, some HSP subtypes, namely SPG7, SPG9A, SPG9B, SPG30, SPG58, and SPG72, can be transmitted in either an autosomal dominant or autosomal recessive manner [1]. Recently, evidence of oligogenic and polygenic contributions in HSP has emerged [11, 12].

AD-HSP is the most common form of HSP, accounting for 70–80% of all cases, followed by AR-HSP in about 30% of cases, and XLR and mitochondrial forms comprise 1–2% of all cases [10]. The most prevalent subtypes of AD-HSP are SPG4 (25%), SPG3A (5%), and SPG31 (3%), while SPG11 (18%), SPG7 (13%), and SPG15 (7%) are the most common subtypes in AR-HSP; however, the prevalence of different HSP subtypes varies among geographical regions [13].

HSP is characterized by significant molecular and clinical heterogeneity, which often makes the diagnosis complex and challenging. Molecular heterogeneity arises from variants in different genes (SPG1–SPG93), resulting in a wide range of manifestations. These heterogeneities can appear as remarkable phenotypic overlaps between HSP and other neurodegenerative disorders [1, 14]. Multiple HSP-causing genes are also implicated in hereditary cerebellar ataxias (*SPG7*, *CAPN1*, *KIF1A*, *SACS*, and *VPS13D*) [15–19], peripheral neuropathies (*BSCL2*, *SPG11*, *MARS1*, *REEP1*, and *RNF170*) [20–24], amyotrophic lateral sclerosis (ALS) (*ERLIN1*, *ERLIN2*, *SPG11*, *ALS2*, and *BSCL2*) [20, 25–27], Parkinson’s disease (PD) (*SPG11*, *ATP13A2*, and *UCHL1*) [28–30], and neurodegeneration with brain iron accumulation (NBIA) (*FA2H*, *PLA2G6, C19ORF12*, and *ATP13A2*) [31].

Due to the high genetic heterogeneity and the involvement of more than 90 genes, the use of NGS technologies, particularly whole-exome sequencing (WES), is highly recommended to improve the genetic diagnosis of HSP [9]. Employing WES allows the identification of known and novel disease-causing genes [32]. Overall, the detection rates of WES in AD-HSP and AR-HSP are 56.7% and 55.5%, respectively, while the detection rate is 21.2% in sporadic forms [33]. Despite the effectiveness of WES in detecting disease-causing variants, approximately 50% of HSP cases remain genetically unresolved [34, 35]. Owing to technical limitations, WES cannot efficiently detect copy number variations (CNVs), which have been previously reported in HSP, especially in the *SPAST, ATL1, SPG11*, and *SPG7* genes [36–38]. Therefore, using a complementary technique such as multiplex ligation-dependent probe amplification (MLPA) alongside WES is recommended to improve the diagnostic rate [39].

In the present study, we conducted a comprehensive genetic and clinical analysis of an Iranian cohort of 103 families, clinically suspected of having HSP. Genetic analysis was performed using a combination of WES and MLPA. The former enabled us to identify disease-causing variants in this cohort, while the latter increased the detection rate by investigating large deletions/duplications missed by WES. It is worth noting that, until 2017, no comprehensive analysis of the genetic and clinical characteristics of HSP patients in Iran had been performed. Therefore, to address this gap, a systematic investigation of HSP was initiated at the Genetics Research Center (GRC) of the University of Social Welfare and Rehabilitation Sciences (USWR) about 8 years ago. Some of the results have been published over the years [40–54], but the rest have not been reported. Here, all obtained data are reported together to speculate the overall genetic landscape of the disease in Iran and to propose a diagnostic framework for evaluating these patients. Our findings contribute to a better understanding of the genetic architecture, subtype distribution, and phenotypic spectrum of HSP in Iran.

## Methods

### Ethical approval

This study received ethical approval from the Ethics Committee of the USWR and the National Institute for Medical Research Development in Iran (IR.USWR.REC.1397.55, IR.USWR.REC.1400.053, and IR.NIMAD.REC.1397.299), and was conducted in accordance with the Declaration of Helsinki. Written informed consent was obtained from all participants or their guardians. All personal and medical information was anonymized to protect the participants confidentiality.

### Subjects

A total of 103 Iranian families suspected of HSP were recruited between 2017 and December 2024 from the Neurology Department of Hazrat Rassoul Hospital, affiliated with Iran University of Medical Sciences, and the Neuromuscular Research Center of Shariati Hospital at Tehran University of Medical Sciences, two major referral centers in Iran. The recruitment was unbiased, and patients were included regardless of sporadic or familial status, the pattern of inheritance, ethnicity, gender, clinical subtype (pure or complicated), and age at onset (AAO) of the disease. Considering that the patients in this cohort were collected and genetically examined over a period of eight years, 27 families have been reported in distinct publications after reaching a final genetic diagnosis, as either case reports or small-scale studies [40–54]. To provide a comprehensive overview of the genetic landscape and phenotypic spectrum of HSP in Iran, all 103 probands were investigated together in this study, as a single cohort.

### Clinical and paraclinical evaluations

Data were collected on various demographic and clinical variables, including sex, age, parental consanguinity, family history of recurrence, AAO of initial symptoms, disease duration, lower-limb spasticity and weakness, and bladder dysfunction. Additional clinical symptoms assessed included ID/cognitive deficits (assessed using Standardized Mini-Mental State Examination; [SMMSE]), ataxia, extrapyramidal involvement, dysphagia, dysarthria, seizures, tremor, distal amyotrophy, scoliosis, peripheral neuropathy, spasticity and weakness of upper limb, hearing impairment, and eye abnormalities. The Spastic Paraplegia Rating Scale (SPRS), a validated disease-specific outcome measure, was employed [55]. Other investigations, including brain magnetic resonance imaging (MRI), electrodiagnostic (EDX) tests, and routine laboratory tests, were also performed.

### Genetic analysis

DNA was extracted from peripheral blood using a standard salting-out procedure. The SureSelect V6-Post Kit was utilized to enrich the exonic regions of the proband’s DNA samples. WES was performed using the Illumina HiSeq 2500/4000 systems (Illumina, CA, USA), and the quality of raw data was evaluated by FastQC. Sequences were aligned to the human reference genome (UCSC GRCh37/hg19), and variant calling was performed using SAMTools, Picard, and the Genome Analysis Toolkit (GATK). Initial filtering processes were used to exclude intergenic, UTR, intronic variants, leaving only exonic, splice site, and exonic/splicing variants. Single nucleotide polymorphisms (SNPs) with a reported minor allele frequency (MAF) exceeding 0.01 from various databases -including the 1000 Genomes, ENSEMBL, NHLBI Exome Sequencing Project, Trans-Omics for Precision Medicine program, Genome Aggregation Database, Healthy Exomes database, Greater Middle East Variome Project, Almena, VarCards database, ABraOM, and Iranome database (comprising ~1200 unrelated Iranian control individuals)- as well as those identified in internal exome data from over 200 unrelated Iranian individuals without neurological conditions, were excluded from the analysis.

Subsequently, homozygous and heterozygous variants were analyzed according to familial inheritance patterns, followed by assessment of variants in genes associated with HSP and other neurodegenerative disorders, including ALS, NBIA, metabolic and mitochondrial disorders, PD, ataxia, and peripheral neuropathy.

To identify potential CNVs within established HSP genes, namely *SPAST*, *SPG11*, and *ATL1*, using WES data, we initially employed GermlineCNVCaller along with the Annotation and Ranking of Human Structural Variations (AnnotSV) [42]. Additionally, in patients with no detected single nucleotide variants (SNVs) or CNVs, potential repeat expansions were assessed using ExpansionHunter software. The results were then visualized utilizing the STRipy (https://stripy.org/) website.

In families without variants in known genes, a search for a potential novel gene was conducted, with a focus on gene function and interaction with previously known genes. These genes were ultimately submitted to GeneMatcher to identify, if possible, additional cases carrying variants in the same genes.

### In silico analysis of variants

Various in silico tools were employed to predict the potential pathogenic effects of SNVs on encoded proteins, including SIFT, PROVEAN, Polyphen-2 HVAR, LRT, MutationTaster, Mutation Assessor, GERP, FATHMM, PhyloP100, and the Combined Annotation Dependent Depletion (CADD: https://cadd.gs.washington.edu/). Finally, InterVar (https://wintervar.wglab.org/), VarSome (https://varsome.com/), Franklin (https://franklin.genoox.com/clinical-db/home), and GeneBe (https://genebe.net/) were utilized to classify variants into five categories (pathogenic, likely pathogenic, variants of uncertain significance [VUS], likely benign, and benign) according to the American College of Medical Genetics and Genomics (ACMG) guidelines [56].

### Screening of the candidate variants

Validation and co-segregation analyses for candidate variants associated with the disease were performed using polymerase chain reaction (PCR), followed by Sanger sequencing. Sequencing was conducted utilizing ABI BigDye terminator chemistry and analyzed on an ABI 3130 Genetic Analyzer (Applied Biosystems, Foster City, CA). Primer sequences are available upon request.

To confirm an identified CNV in one patient, MLPA was performed using the SALSA® MLPA® Probemix P165-C3 HSP mix-1 (MRC-Holland, Amsterdam, The Netherlands), following the manufacturer’s protocol.

To confirm the identified repeat expansion in another patient, we employed fluorescence-based PCR to amplify the repeat region. The size of the amplified product from fluorescence-based PCR was analyzed by capillary electrophoresis on an ABI sequencer (Applied Biosystems).

## Results

**Fig. 1:**
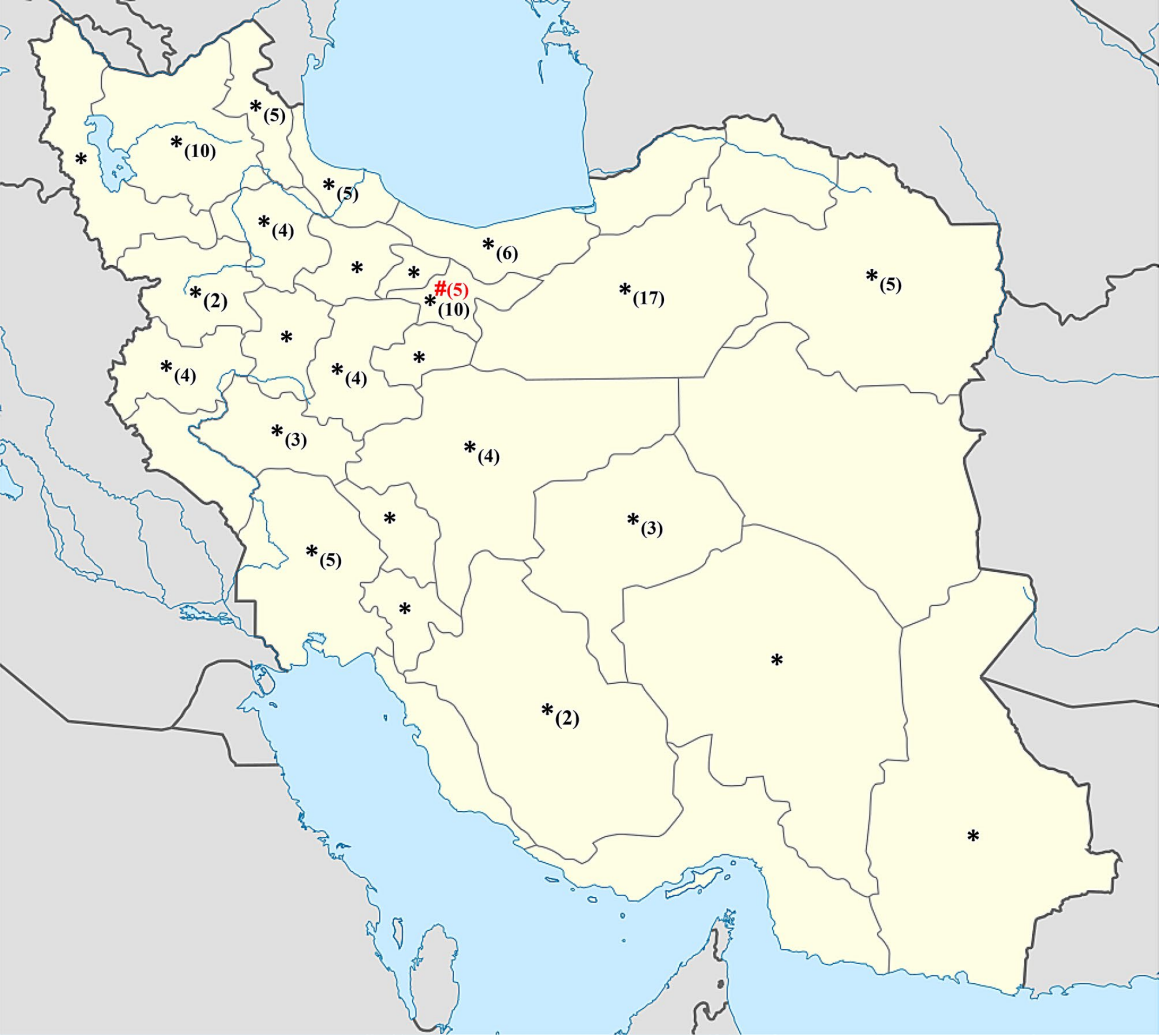
Geographic distribution of 103 unrelated Iranian probands on the map. The symbol # indicates individuals who reside in Tehran, but whose exact place of birth is unknown

To the best of our knowledge, the present study reports the largest cohort of HSP patients from Iran, and possibly from the Middle East. In this study, a cohort of 103 unrelated Iranian families suspected of HSP from all over Iran (Fig. 1) was evaluated. It is imperative to acknowledge once again that a few of the cases presented here have been reported previously [40–54]. These studies were referenced in the “Methods” section and Supplementary file S1.

### Demographic information

Over an eight-year period, 103 unrelated families were enrolled in this study, comprising 220 affected individuals (Supplementary file S1). These families belonged to different Iranian ethnic groups, including Persian, Azeri, Kurd, Arab, Lur, Turkmen, Baloch, Mazani, and Gilak. Regarding inheritance patterns, 46 probands were classified as sporadic, 43 families exhibited AR inheritance, one of them had both HSP (AR) and neuropathy (AD) simultaneously, and 12 families showed AD inheritance. One family demonstrated an XLR pattern, and for one genetically undiagnosed proband, the mode of inheritance remained unclear, possibly either AD or AR. Consanguinity was present in 77.6% (80/103) of the families.

The probands cohort exhibited an approximately equal gender distribution (50.5% female vs 49.5% male). The mean current age of the probands was 31.67 ± 14.71 years (range: 9–75 years). The mean AAO for probands was 14.52 ± 12.59 years, with onset ranging from infancy to 54 years. No significant differences were observed in the mean AAO (males: 12 ± 13.14 years and females: 14 ± 11.89 years) or disease duration (males: 19 ± 13.6 years and females: 16 ± 8.32 years) between male and female probands.

### Clinical and paraclinical findings

Gait disturbances (tiptoe walking, spastic and ataxic gait, and frequent falls) were the main initial symptoms observed across the probands of the cohort. Lower-limb spasticity was observed in all probands, while lower-limb weakness was absent in only one proband (HSP144). Nearly all probands exhibited increased DTRs in their lower limbs, except one proband (HSP159), in whom all DTRs were absent except the triceps reflex. The Babinski sign was assessed in only 60 probands; among them, 54 showed a positive Babinski sign, while it was negative in six probands, including the probands of HSP104, HSP106, HSP129, HSP134, HSP155, and HSP214 families. Urinary dysfunction and foot deformities were each observed in 39.8% (41/103) of all probands.

Based on the genetic findings, the probands with their corresponding genes were categorized into three main groups (Table 1) as follows.

**Table 1:** Classification of the probands with their identified genes into three main groups in our cohort

Group	Disease categories	Genes	# Families	# Genes	# Variants
A	Linked to 1 out of 93 HSP subtypes (SPG1–SPG93) based on OMIM database	*L1CAM (SPG1), ATL1 (SPG3A), SPAST (SPG4), CYP7B1 (SPG5A), SPG7 (SPG7), KIF5A (SPG10), SPG11 (SPG11), HSPD1 (SPG13), ZFYVE26 (SPG15), ERLIN2 (SPG18), FA2H (SPG35), C19orf12 (SPG43), GJC2 (SPG44), GBA2 (SPG46), AP4S1 (SPG52), CYP2U1 (SPG56), ERLIN1 (SPG62), ENTPD1 (SPG64), RAB3GAP2 (SPG69), CAPN1 (SPG76), HPDL (SPG83)*	64	21	54
B	Related to other neuromuscular/neurodegenerative diseases	*KIF1B, MFN2, MTPAP, CACNA1A, SPTBN2, GCH1, SPEG, MYH2, PRDM8, COQ7, RNASEH2B, ALS2*	14	12	13
C	Putative novel genes for HSP	*SEMA3A, KCNJ14, EMP3, NMNAT1*	3	4	4
Total	-	-	81	37	71

HSP: hereditary spastic paraplegia, SPG: spastic paraplegia genes, OMIM: Online Mendelian Inheritance in Man

#### Group A

**Fig. 2:**
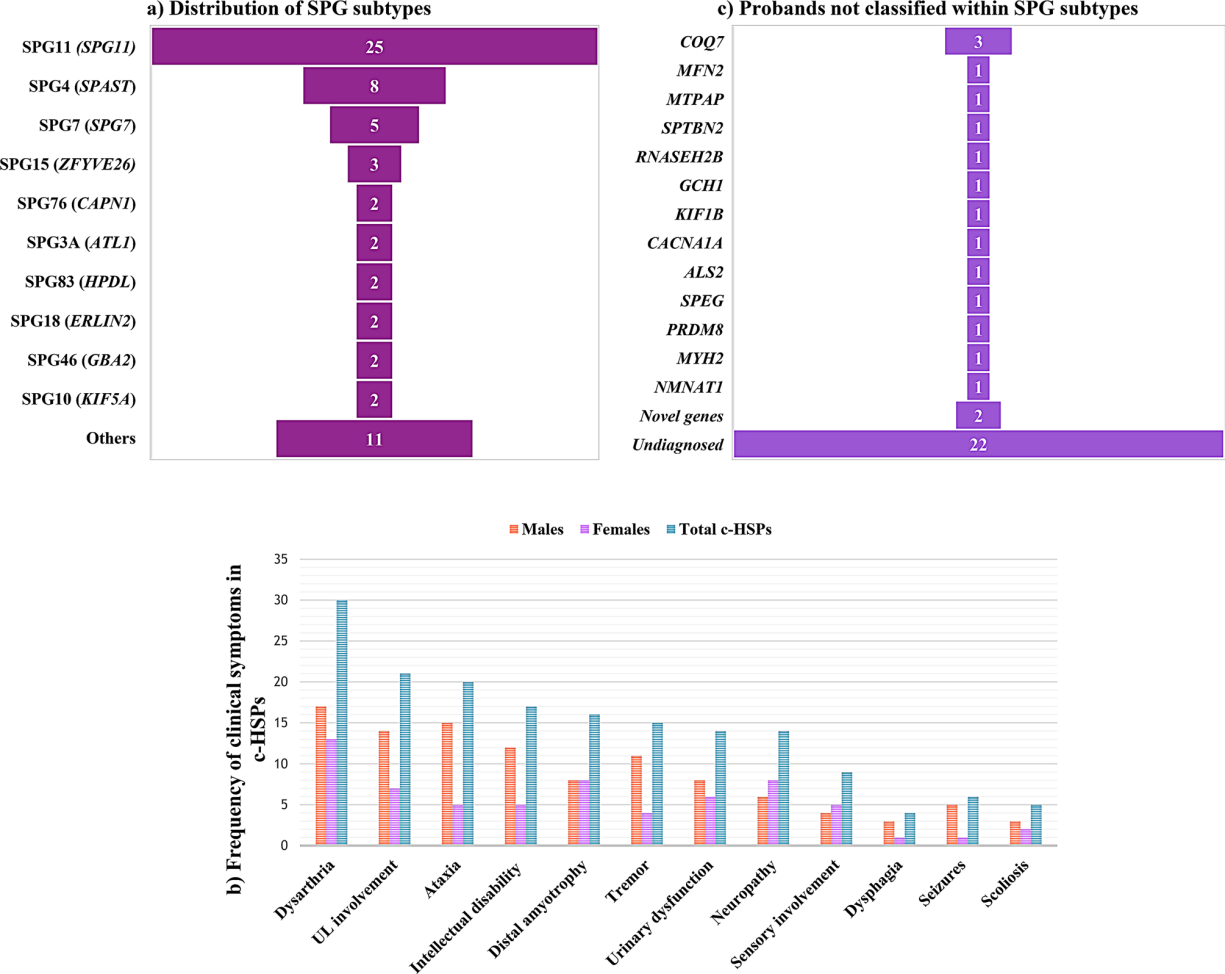
**a**) Distribution of SPG subtypes; **b**) Frequency of clinical symptoms in c-HSP probands; **c**) Probands not classified within SPG subtypes

62.1% (64/103) of the probands were categorized into a distinct subtype of HSP (SPG1–SPG93) (Fig. 2a). Of these, 21.8% (14/64) showed the pure form of the disease, whereas 78.2% (50/64) manifested a complicated form of HSP (Table 2a). P-HSP probands, as expected, exhibited only the typical symptoms of the disease, with a mean AAO of 15.10 ± 13.38 years (range: 1–48 years). In contrast, c-HSP probands, in addition to these symptoms, manifested several other features. The most common symptoms in the c-HSP probands were dysarthria, observed in 60% (30/50), followed by upper-limb involvement in 42% (21/50), ataxia in 40% (20/50), ID in 34% (17/50), distal amyotrophy in 32% (16/50), tremor in 30% (15/50), urinary dysfunction in 28% (14/50), and neuropathy in 28% (14/50). The mean AAO of the c-HSP group was 14.89 ± 10.15 years (range: 0–54 years) (Supplementary file S1, Fig. 2b and Table 2a).

**Table 2:** **a**. Overall and demographic features of all Iranian HSP subtypes in this study. **b**. Overall and demographic features of all Iranian suspected HSP cases without variants in known HSP genes in this study

**(a)**	**Total probands with a HSP subtype**	**p-HSP**	**c-HSP**
	64	14 (21.9)	50 (78.1)
Gender (%)			
-Male	35 (54.7)	5 (35.7)	30 (60)
-Female	29 (43.3)	9 (64.3)	20 (40)
Present age (mean and range; yrs)	31.06 +/- 13.55 (9–75)	29.14 +/- 15.95 (9–56)	31.6 +/- 12.93 (10–75)
Age at onset (mean and range; yrs)	14.93 +/- 10.82 (0–54)	15.1 +/- 13.38 (1–48)	14.89 +/- 10.15 (0–54)
Conasanguinity	50 (78.1)	10 (71.4)	40 (80)
# Multi-affected families	35 (54.7)	4 (28.6)	31 (62)
The mode of inheritance (%)			
-Autosomal recessive	27 (42.2)	2 (14.3)	25 (50)
-Autosomal dominant	7 (10.9)	2 (14.3)	5 (10)
-X-linked	1 (1.5)	0	1 (2)
-Sporadic	29 (45.3)	10 (71.4)	19 (38)
**(b)**	**Total probands**	**p-HSP**	**c-HSP**
Number of cases (%)	39	7 (17.9)	32 (82.1)
Gender (%)			
-Male	16 (41)	3 (42.8)	13 (40.6)
-Female	23 (59)	4 (57.2)	19 (59.4)
Present age (mean and range; yrs)	32.64 +/- 16.54 (10–65)	20.42 +/- 12.89 (10–41)	35.31 +/- 16.19 (10–65)
Age at onset (mean and range; yrs)	13.84 +/- 15.18 (1–51)	7.57 +/- 6.26 (2–18)	15.21 +/- 16.25 (1–51)
Conasanguinity	30 (76.9)	6 (85.7)	24 (75)
# Multi-affected families	22 (56.4)	5 (71.4)	17 (53.1)
The mode of inheritance (%)			
-Autosomal recessive	16 * (41)	4 (57.14)	12 * (37.5)
-Autosomal dominant	5 * (12.8)	1 (14.3)	4 * (12.5)
-X-linked	0	0	0
-Sporadic	17 (43.6)	2 (28.6)	15 (46.9)

HSP: hereditary spastic paraplegia, yrs: years, p-HSP: pure-HSP, c-HSP: complicated-HSP

*The mode of inheritance is ambigous in one family (It could be autosomal recessive or dominant)

#### Groups B and C

In contrast, 39 probands (37.9%) did not belong to any of the main HSP subtypes. This group of patients either had variants in genes related to other neuromuscular/neurodegenerative diseases (Group B; 14/39) or, candidate novel genes (Group C; 3/39) or remained genetically undiagnosed (22/39) (Fig. 2c). Among them, 17.9% (7/39) presented p-HSP (their mean AAO was 7.57 ± 6.26 years; range: 2–18 years), whereas 82.1% (32/39) manifested c-HSP (their mean AAO was 15.21 ± 16.25 years; range: 1–51 years) (Table 2b). Interestingly, in proband HSP155, variants in both *ERLIN2* and *MFN2* coexisted.

### Electromyography (EMG) and nerve conduction study (NCS) findings

42/103 probands had no reliable EMG/NCS data. Among the 61 remaining ones, EMG and NCS abnormalities were detected in 45.9% (28/61) of the probands. Peripheral neuropathy was the predominant abnormality with a frequency of 71.4% (20/28), followed by anterior horn cell disease in 25% (7/28). As expected, probands with p-HSP manifested no involvement in EMG/NCS, and such abnormalities were observed only in c-HSP probands (Supplementary file S2).

### Neuroimaging findings

**Fig. 3:**
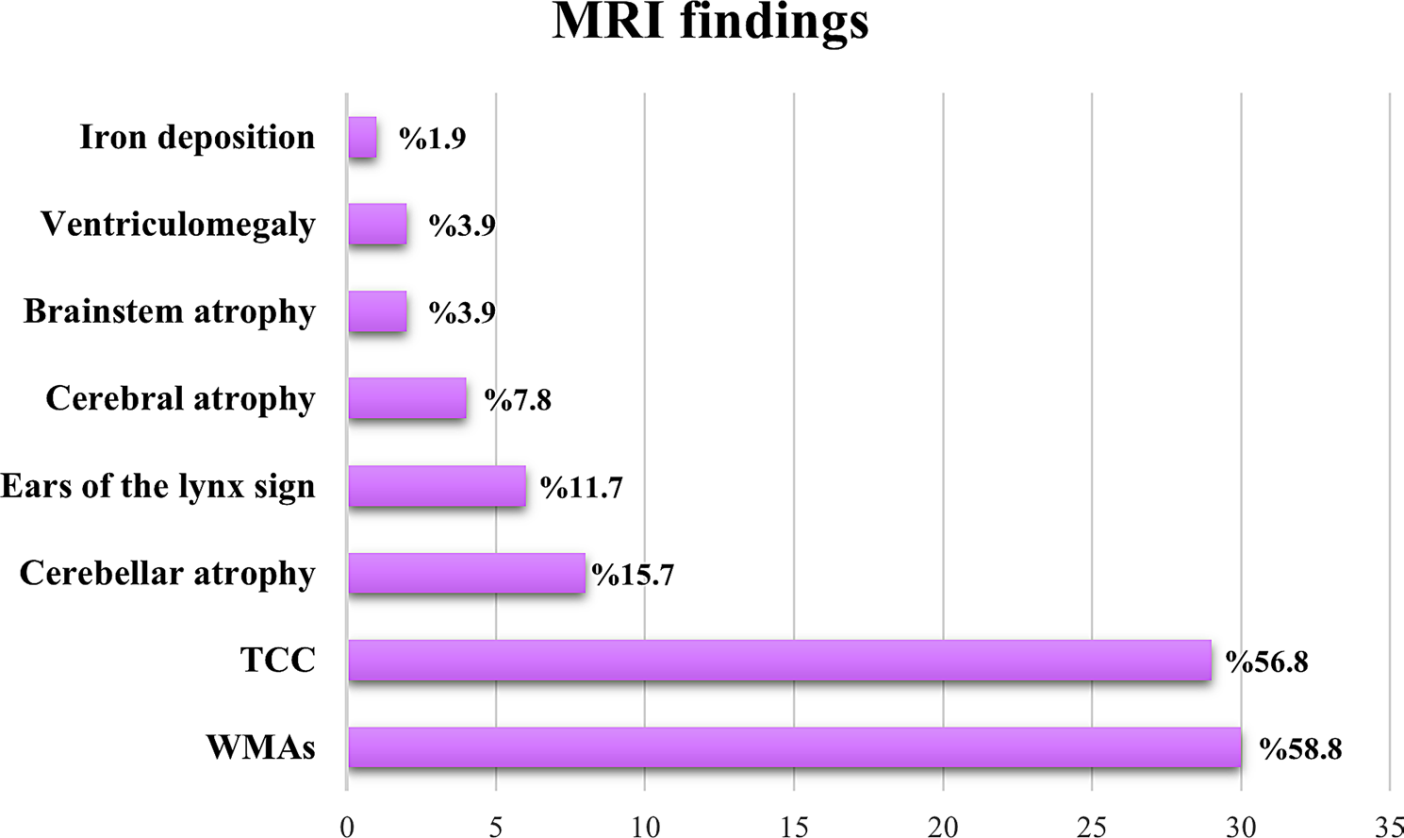
Frequency of MRI abnormalities observed in our HSP cases. It should be noted that 33 out of the 84 probands showed no MRI abnormalities; therefore, the present analysis was conducted based on the 51 probands with abnormal MRI findings. TCC: thin corpus callosum, WMAs: white matter abnormalities

MRI of the brain and spine was unavailable for 19 probands. The MRI conducted on the remaining 84 probands revealed the absence of any abnormality in 39% (33/84) of probands. The existence of white matter abnormalities (WMAs) and a thin corpus callosum (TCC), which were the predominant findings in our cohort, were observed in 58.8% (30/51) and 56.8% (29/51) of cases, respectively. Moreover, some additional findings included cerebellar atrophy in 15.7% (8/51), “ears of lynx sign” in 11.7% (6/51), cerebral atrophy in 7.8% (4/51), and brainstem atrophy in 3.9% (2/51) (Supplementary file S3 and Fig. 3).

### Genetic findings

According to WES data evaluation, high sequencing quality was validated for all the probands. Based on the pipeline described in the “Methods” section, SNVs and CNVs were searched for. In total, the primary diagnostic yield across 103 probands was approximately 70.8% (73/103), considering only pathogenic and likely pathogenic variants. Additional analyses and detailed diagnostic categories were summarized in Table 3. Among the genetically solved probands, including those with pathogenic, likely pathogenic, and VUS variants (81/103), the majority of cases were sporadic, accounting for 46.9% (38/81), 39.5% showed autosomal recessive inheritance (32/81), and 12.3% (10/81) demonstrated AD inheritance. Only one family (1.2%; 1/81) exhibited XLR inheritance, carrying a variant in the *L1CAM* gene. Notably, in one proband, two distinct disease-causing variants in the *ERLIN2* and *MFN2* genes were detected, with AR and AD inheritances in his family, respectively (Supplementary file S4). This case was previously reported by our group [45].

**Table 3:** Definitions of diagnostic categories and the corresponding results in our cohort

Diagnostic category	Definition	# Probands	% Probands
Genetically solved probands (only P/LP variants) (primary diagnostic yield)	Probands with identified possible disease-causing variants considering only P/LP variants	73/103	**70.8%**
A. Diagnostic rate of HSP cases	Probands with P/LP variants in known HSP genes (SPG1–SPG93)	59/103	57.2%
B. Diagnostic rate of cases with variants in non-HSP genes	Probands with P/LP variants in non-HSP genes	14/103	13.5%
Total genetically solved probands (P/LP/VUS)	Probands with identified possible disease-causing variants	81/103	78.6%
A. HSP probands with a genetic diagnosis	Genetically solved probands with variants in known HSP- genes (SPG1–SPG93)	64/81	79%
B. Probands with variants in non-HSP genes	Genetically solved probands with variants in genes not related to HSP (non-SPG genes)	17/81	20.9%
− Other neuromuscular/neurodegenerative disorders *	Variants in genes related to other neuromuscular/neurodegenerative disorders	14/17	82.3%
− Putative novel genes	Variants in genes not related to HSP or other neuromuscular/neurodegenerative disorders	3/17	17.6%

P: Pathogenic, LP: likely pathogenic, VUS: varinat of uncertain significance, HSP: hereditary spastic paraplegia, SPG: spastic paraplegia genes, * These including peripheral neuropathy, amyotrophic lateral sclerosis (ALS), coenzyme Q10 deficiency, spinocerebellar ataxia (SCA), spastic ataxia, myoclonus epilepsy, myopathies, and Aicardi-Goutières syndrome (AGS)

**Fig. 4:**
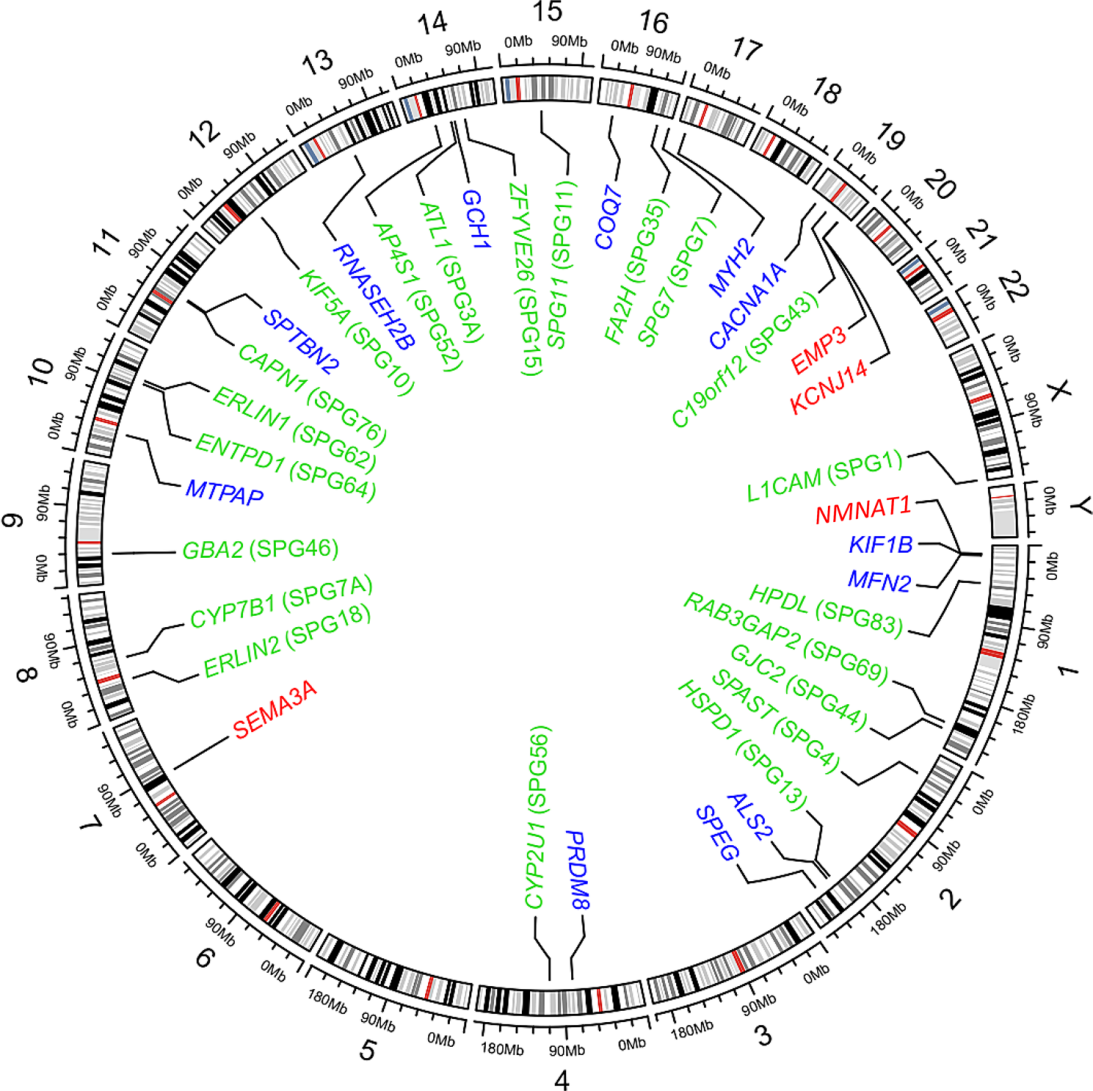
Schematic representation of all identified disease-causing genes in this study. Green-colored genes: known SPG genes, blue-colored genes: genes related to other neuromuscular/neurodegenerative disorders, red-colored genes: novel candidate genes

**Fig. 5:**
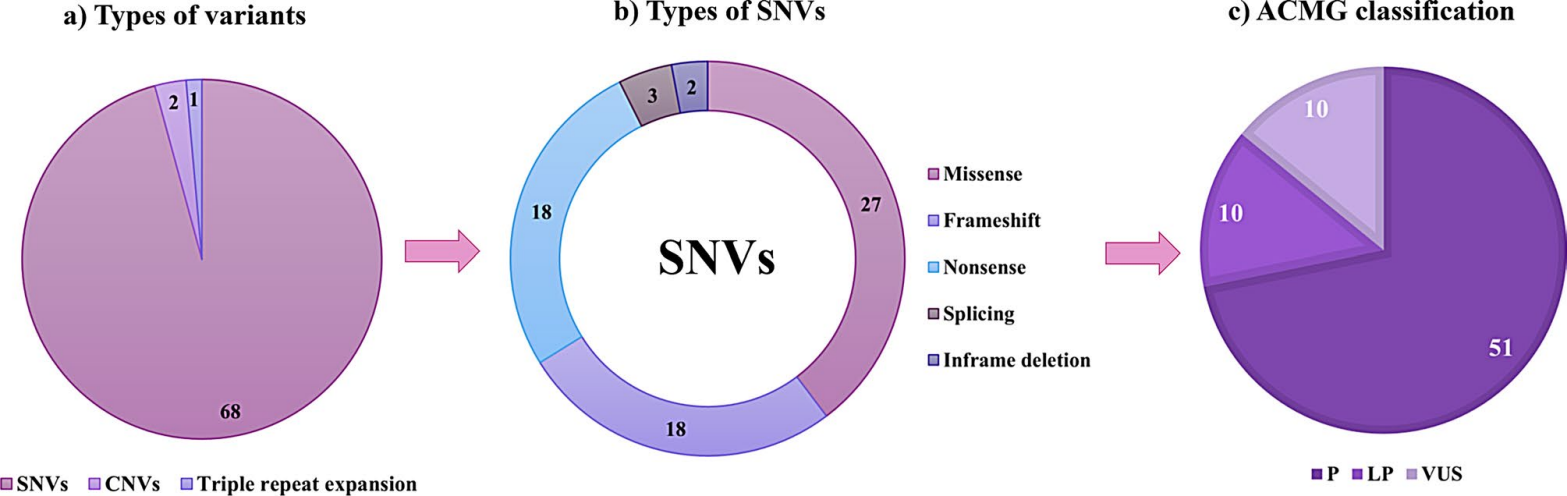
Genetic characteristics of our HSP patients. (**a**) Types of identified variants, (**b**) Types of SNVs, (**c**) ACMG classification of all variants. SNVs: single nucleotide variants, CNVs: copy number variants, ACMG: American College of Medical Genetics and Genomics, P: pathogenic, LP: likely pathogenic, VUS: variant of uncertain significance

In total, 71 variants were identified in 37 distinct genes. These 37 identified genes were categorized into three main groups (Table 1, Fig. 4). All candidate variants were screened in the corresponding families and showed co-segregation with disease. Overall, the majority (95.7%; 68/71) of identified variants in the cohort were SNVs, while CNVs accounted for 2.8% (2/71), and only a single case (1.4%; 1/71) harbored a trinucleotide (CAG) repeat expansion (Fig. 5a). Among the total SNVs, the most frequently observed variants were missense comprising 39.7% (27/68), followed by frameshift in 26.4% (18/68), nonsense in 26.5% (18/68), splicing in 4.4% (3/68), and in-frame deletion in 2.9% (2/68) (Fig. 5b). Both CNVs were large deletions in the *SPAST* gene and confirmed by MLPA. One involved a previously reported single-exon deletion (exon 17, the last exon), while the other was a novel multi-exon deletion spanning exons 1–4. Additionally, out of 103 probands, only one patient carried a 33-repeat CAG expansion in the *CACNA1A* gene. Overall, 85.9% (61/71) of the variants in 37 genes mentioned above were predicted as either pathogenic or likely pathogenic, whereas 14.1% (10/71) were classified as VUS based on the ACMG guidelines (Fig. 5c). Furthermore, 54.9% (39/71) of the detected variants were previously reported, while 45.1% (32/71) were novel variants.

In total, the most frequently observed subtype of HSP was SPG11/*SPG11*, representing 24.2% (25/103) of all probands, followed by SPG4/*SPAST* in 7.7% (8/103). Other frequently mutated genes were *SPG7* (4.8%; 5/103), *SPG15* (2.9%; 3/103), and *COQ7* (2.9%; 3/103). Furthermore, *ATL1*/SPG3A, *KIF5A*/SPG10, *ERLIN2*/SPG18, *GBA2*/SPG46, *CAPN1*/SPG76, *HPDL*/SPG83, and *MFN2*/CMT2A2A were each mutated in 2 out of 103 probands (1.9%). Notably, 11 rare subtypes with only one proband were *L1CAM*/SPG1, *CYP7B1*/SPG5A, *HSPD1*/SPG13, *FA2H*/SPG35, *C19orf12*/SPG43, *GJC2*/SPG44, *AP4S1*/SPG52, *CYP2U1*/SPG56, *ERLIN1*/SPG62, *ENTPD1*/SPG64, and *RAB3GAP2*/SPG69 (Supplementary file S4, Fig. 2a & 2c).

## Discussion

Herein, we present the clinical and genetic data of a cohort of 103 unrelated Iranian families suspected of HSP. Some families included in this cohort (27/103) were previously reported in separate publications, reflecting the long-term, eight-year data collection period of the study. These cases were included to provide a comprehensive overview of the genotypic and phenotypic spectra of HSP in Iran, and their inclusion does not substantially alter subtype frequencies or drive variant recurrence. Overall, our findings highlight the marked genetic heterogeneity of HSP and its overlap with other neurological disorders, emphasizing the value of large cohorts and high-throughput sequencing approaches such as WES for improving molecular diagnosis. Additionally, identifying shared genes and pathways across these overlapping disorders may reveal common pathogenic mechanisms and help guide the development of targeted therapeutic strategies.

**Fig. 6:**
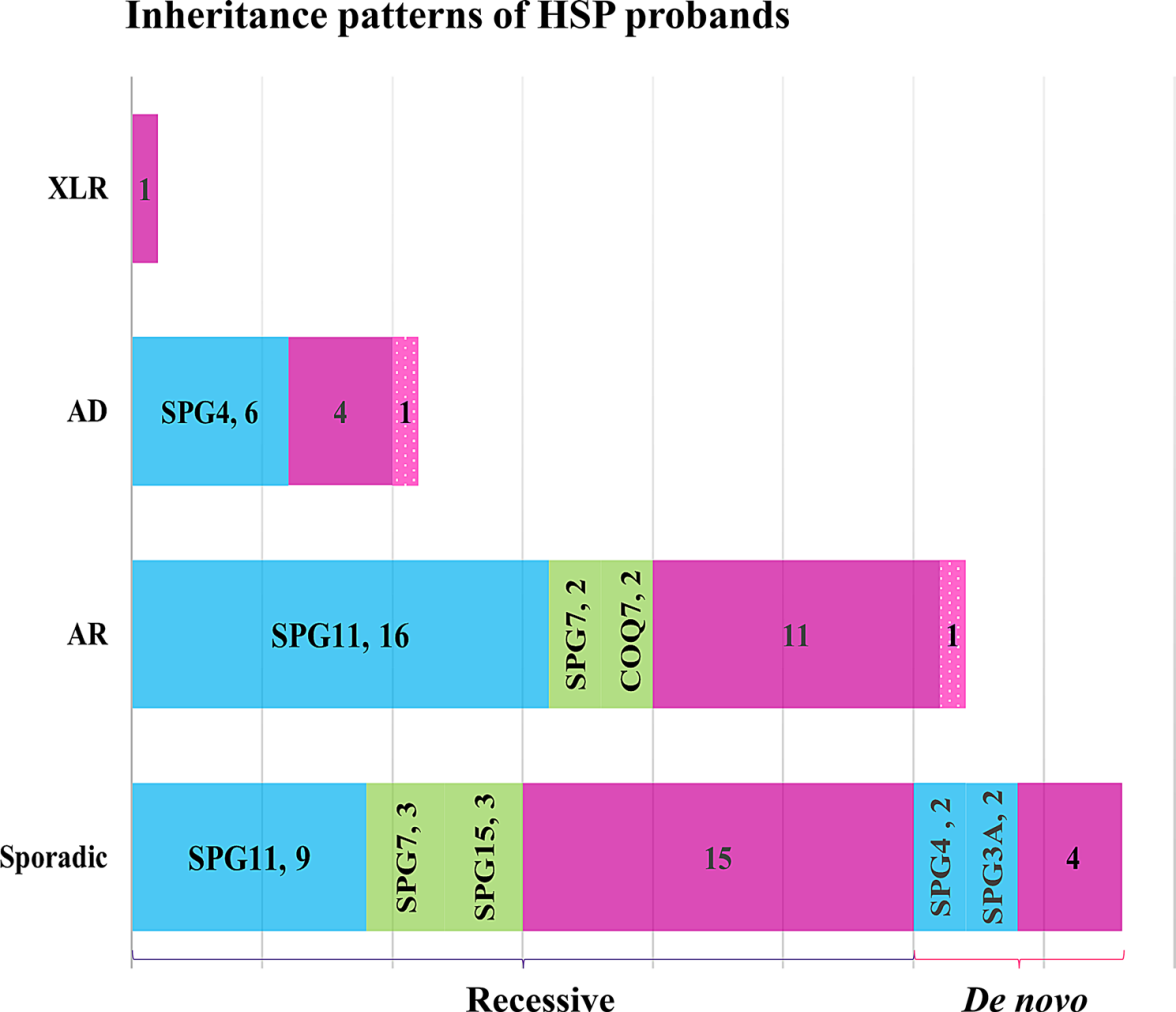
Number of families with different inheritance patterns. The most common and the second most common subtypes are illustrated in blue and green colors, respectively. The pink dotted line represents a family displaying both AD and AR inheritance with two different variants in the *ERLIN2* and *MFN2* genes. XLR: X-linked recessive, AD: autosomal dominant, AR: autosomal recessive

**Fig. 7:**
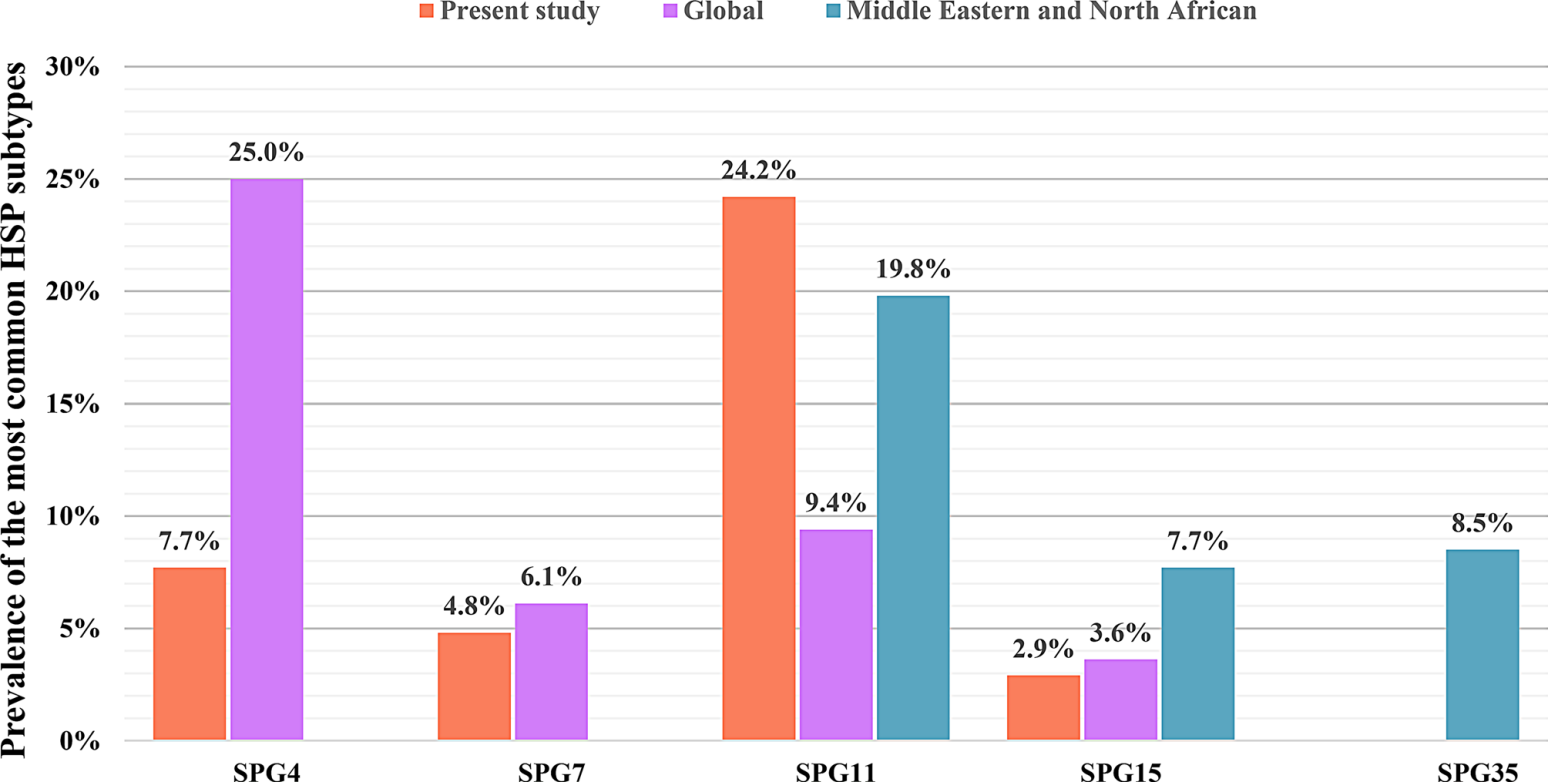
Distribution of major HSP subtypes across different populations. This chart illustrates the prevalence of the most common HSP subtypes in the present study (Iranian probands) compared with global and Middle Eastern/North African populations

As explained above, 21 distinct HSP subtypes were identified across 64 probands within Group A (Table 1). The most frequent HSP subtypes were SPG11 (24.2%), SPG4 (7.7%), SPG7 (4.8%), and SPG15 (2.9%) (Fig. 2a). It should be noted that additional subtypes, including SPG9/*ALDH18A1*, SPG50/*AP4M1*, SPG54/*DDHD2*, SPG57/*TFG*, SPG85/*RNF170*, and SPG87/*TMEM63C*, have been rarely reported in the limited studies of HSP conducted within the Iranian population [57]. The high frequency of SPG11 in our cohort (39.1% of Group A probands and 50% of AR-HSP cases; Fig. 6) may be attributable to the high rate of consanguineous marriages in Iran, as has also been reported in Middle Eastern and North African countries [57] (Fig. 7). In comparison, the frequency of *SPG11* variants reported in other populations is considerably lower, ranging from 2% in Italy (Pisa/Tuscany) to 11% in China among all HSP cases, and 20–30% among AR-HSP cases [58–60]. Meanwhile, SPG4 was the most common HSP subtype in most European cohorts, particularly among AD-HSP cases. For example, a Canadian study reported that SPG4 accounted for up to 48% of all HSP cases [61–63]. In contrast, in our cohort, SPG4 was the second most common HSP subtype (7.7%) (Figs. 6 and 7). Notably, all familial SPG4 cases in our study showed genetic anticipation; a phenomenon previously reported in several studies [39, 49, 64].

The most frequent variant identified was c.3075dup in *SPG11*, detected in 10 families and located within a poly-A tract, suggesting a potential mutational hotspot. Other recurrent variants included c.3036C > A, p.Tyr1012* in *SPG11*, c.233T > A, p.Leu78* in *SPG7*, and c.1496 G > A, p.Arg499His in *SPAST*. These findings highlight important considerations for gene prioritization during genetic screening.

Among the 14 probands in Group B, who carried 13 variants in 12 genes, the most frequently mutated genes were *COQ7* (2.9%; 3/103) and *MFN2* (1.9%; 2/103) (Fig. 2c).

### Clinical findings of the Group A

**Fig. 8:**
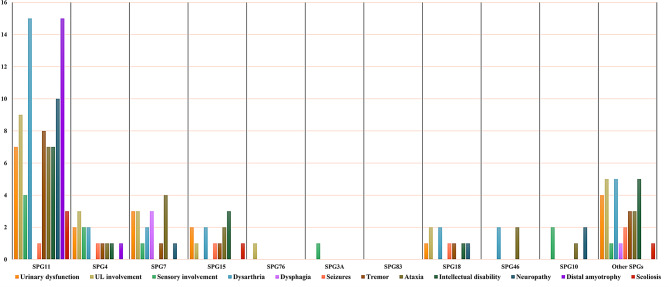
Frequency of the most prevalent clinical features in SPG subtypes

Among the 64 probands within Group A, 14 (21.9%) were diagnosed with p-HSP, while the remaining 50 (78.1%) had c-HSP (Table 2a). In the p-HSP group, lower-limb spasticity was observed in all probands (100%), whereas urinary dysfunction and sensory impairment were reported in 35.7% (5/14) and 14.3% (2/14) of cases, respectively, consistent with previous reports [13, 65]. Among c-HSP probands, dysarthria, upper-limb involvement, and cerebellar ataxia were the most common features, occurring in 60% (30/50), 42% (21/50), and 40% (20/50) of probands, respectively. Dysarthria and upper-limb involvement were particularly common among SPG11 probands, occurring in 60% (15/25) and 36% (9/25), respectively, whereas upper-limb involvement was also frequent in SPG4 and SPG7. The relatively high frequency of ataxia further supports the known clinical and genetic overlap between HSP and hereditary ataxias (Figs. 2b, 8 and Table 4, and Supplementary file S1).

**Table 4:** Summary of key clinical and paraclinical features across the most frequent c-HSP subtypes in this study

	SPG11 (25)	SPG4 (8)	SPG7 (5)	SPG15 (3)	SPG10 (2)	SPG18 (2)
Dysarthria	15/25 (60%)	2/8 (25%)	2/5 (40%)	2/3 (66.6%)	0	2/2 (100%)
Upper-limb involvement	9/25 (36%)	3/8 (37.5%)	3/5 (60%)	1/3 (33.3%)	0	1/2 (50%)
Ataxia	7/25 (28%)	1/8 (12.5%)	4/5 (80%)	2/3 (66.6%)	1/2 (50%)	0
Intellectual disability	7/25 (28%)	1/8 (12.5%)	0	3/3 (100%)	0	1/2 (50%)
Distal amyotrophy	15/25 (60%)	1/8 (12.5%)	0	0	0	0
Peripheral neuropathy	10/25 (40%)	0	1/5 (20%)	0	2/2 (100%)	1/2 (50%)
WMAs	14/25 (56%)	1/8 (12.5%)	0	2/3 (66.6%)	0	0
TCC	22/25 (88%)	0	0	3/3 (100%)	0	0
Ears of lynx	3/25 (12%)	0	0	2/3 (66.6%)	0	0

WMAs: white matter abnormalities, TCC: thin corpus callosum, c-HSP: complicated HSP

Another notable feature observed in our c-HSP probands was ID, which was present in 34% (17/50) of cases (Table 4, Fig. 2b). Contrary to previous studies, which indicate a relatively high frequency of ID in SPG4 and SPG7 patients, our findings were not compatible with previous reports (Table 4) [13].

Distal amyotrophy, a sign of lower motor neuron (LMN) involvement, was observed in 16 of 50 c-HSP cases. Remarkably, 93.7% (15/16) of these cases were SPG11 cases, further supporting the strong association between SPG11 and LMN involvement [66].

**Fig. 9:**
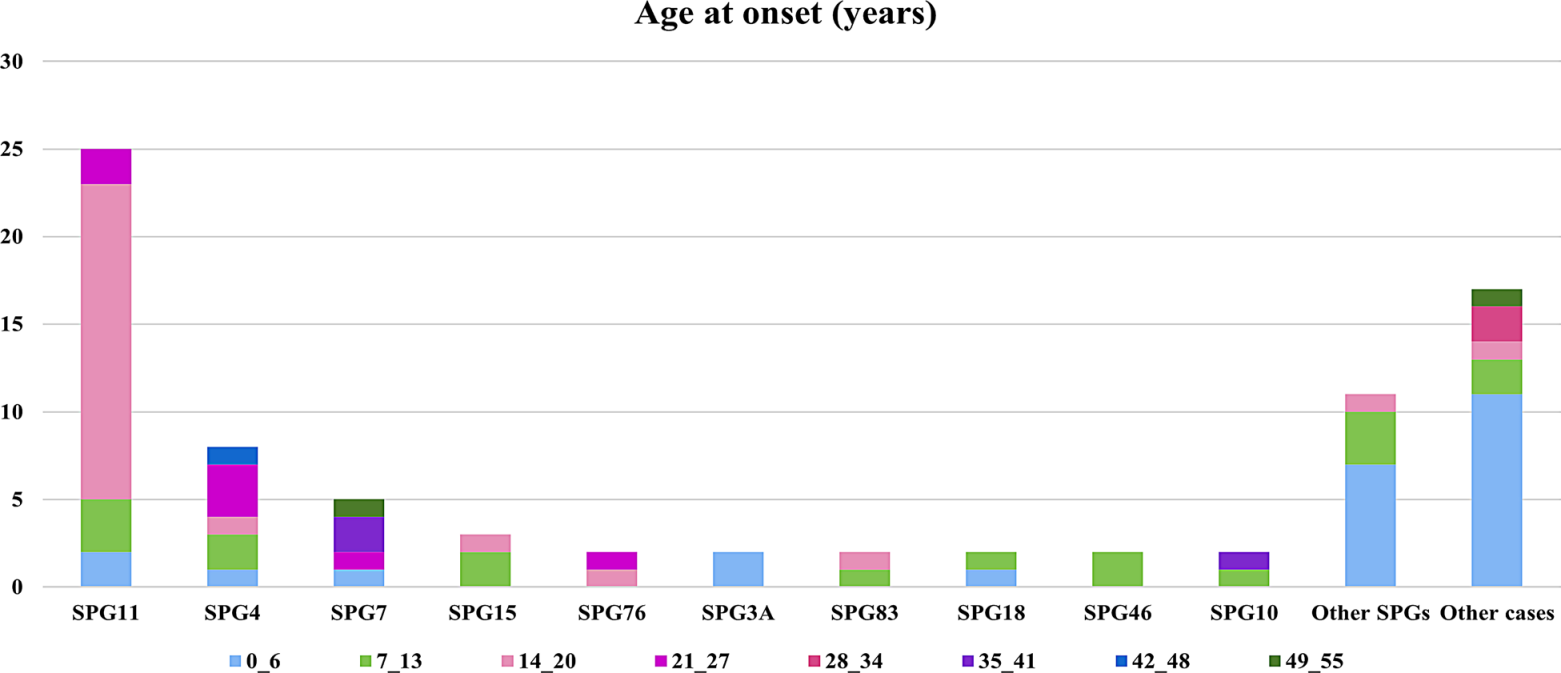
Age at onset of all HSP probands. Different age ranges (x-axis) are represented by various colors, while the y-axis indicates the number of probands

Clinically, HSP can present at any age; however, in our cohort, it appeared more frequently in probands aged 10 years and younger. The AAO varied across different HSP subtypes (Fig. 9), among unrelated families, and even within affected individuals of each family. Overall, the mean AAO for Group A probands was 14.93 ± 10.82 years. In AD-HSP probands (all probands with a heterozygous variant), the average AAO was 16.88 ± 14.59 years, ranging from 1 to 48 years, with particularly early onset in SPG3A cases, as previously reported [13]. For AR-HSP probands (all probands with a homozygous variant), the mean AAO was 14.65 ± 9 years, ranging from 1 to 54 years. The single proband with XLR inheritance exhibited disease onset at four years of age.

Among SPG11 probands, the most prevalent subtype in our cohort, the mean AAO was 15.3 ± 4.69 years. Except for three individuals, all SPG11 probands exhibited disease onset before the age of 20. In comparison, the AAO reported for HSP subtypes in other large cohorts appears higher; for instance, Cao et al. and Shi et al. reported mean AAOs of 23.03 ± 16.37 and 28.31 ± 15.92 years, respectively [39, 67].

### Electrodiagnostic findings of the Group A

In contrast to p-HSP, a spectrum of abnormalities was observed in EMG/NCS of c-HSP probands, which are summarized below (Supplementary files S2).

Neuropathy was a notable feature in c-HSP, occurring in 28% (14/50) of probands (Table 4). Chronic anterior horn cell disease, another type of LMN involvement, was detected in 16% (4/25) of SPG11 probands via EMG-NCS, highlighting a broader neurodegenerative pattern in these cases. Although HSP is primarily characterized by UMN involvement, LMN abnormalities were observed in a subset of patients, highlighting the diagnostic complexity of these cases.

### Neuroimaging findings of the Group A

Many HSP subtypes do not cause significant changes on brain MRI, whereas some subtypes are associated with detectable abnormalities, including WMAs, TCC, and cerebellar or cerebral atrophy. These MRI findings can provide supportive diagnostic clues in c-HSP forms like SPG11, SPG15, and SPG35, where abnormalities are more frequently observed and can help differentiate subtypes with overlapping clinical features [39].

WMAs were observed in 42% (21/50) of c-HSP probands. Among these, 66% (14/21) were SPG11 (Table 4). TCC has been reported in many HSP subtypes, especially SPG11. In our cohort, TCC was observed in 26 of 50 c-HSP probands (Table 4). The “ears of lynx” sign, an MRI abnormality that is frequently seen in SPG11 and SPG15 subtypes [68], was observed in 5 of 50 c-HSP cases (10%), including two SPG15 and three SPG11 probands (Table 4).

Another notable MRI finding in HSP probands was brain iron accumulation. In this study, pathogenic variants were identified in two NBIA-associated genes, *FA2H* and *C19ORF12,* which are now also recognized as main HSP-related genes. The proband carrying the *FA2H* variant (HSP212), exhibited mild brain iron accumulation, whereas the patient with the *C19ORF12* variant (HSP188), had a normal MRI without iron deposition. These findings are consistent with previous reports linking *FA2H* variants to SPG35 with brain iron accumulation [69] and *C19ORF12* to SPG43 without iron deposition [70].

Our findings indicate that MRI abnormalities were not specific to any single HSP subtype. Hence, MRI alone is insufficient for differential diagnosis, highlighting the pivotal role of genetic analysis in achieving a definitive diagnosis (Supplementary file S3 and Fig. 3).

### Group B: genes associated with neuromuscular/neurodegenerative disorders

All 14 probands within Group B (Table 1) were initially referred to us as suspected HSP cases. Among these probands, one (7.1%) was classified as p-HSP, whereas the remaining 13 (92.8%) were diagnosed with c-HSP. The mean AAO was 8.5 ± 13.94 years. Among them, 10 probands carried homozygous variants; nine manifested the disease before the age of 5 years (Fig. 9), while only one presented at 51 years. The remaining four probands, carried heterozygous variants (Supplementary Files S1 and S4). In this group, variants in most genes were observed in single families (except for *MFN2* and *COQ7*), and all variants segregated strongly within families. Additionally, these genes were further subdivided into three subgroups, which reflect the genetic and clinical complexity of our cohort.

#### Subgroup 1 (expansion of the HSP genetic spectrum; *COQ7, GCH1,* and *RNASEH2B*)

It consists of putative genes proposed to expand the disease’s gene spectrum. In some recent studies, they have been rarely reported as being linked to HSP [44, 71–77]. For example, a shared variant in *COQ7*, a gene involved in coenzyme Q10 biosynthesis, was detected in three unrelated families (HSP130, HSP149, and HSP 205) presenting with c-HSP accompanied by hearing loss, suggesting a possible founder effect [44]. Variants in this gene have also recently been linked to pure HSP [71, 77]. Additionally, the links between HSP and other CoQ10-related genes (*COQ4* and *COQ9*) [78–81] have been reported in recent studies [82]. Similarly, variants in *GCH1* and *RNASEH2B* genes, classically linked to DOPA-responsive dystonia and Aicardi-Goutières syndrome, respectively [83, 84], were also identified in the probands HSP187 and HSP144, respectively, presenting with c-HSP phenotypes; variants in both genes have rarely been reported in HSP cases [74, 76, 85, 86].

Overall, further investigations and functional validation are necessary, and definitive assignment of a causal role for subgroup 1 genes in HSP pathogenesis remains premature.

#### Subgroup 2 (phenotypic overlap; *ALS2* and *MTPAP*)

Variants identified in these genes represent phenotypic overlap rather than classical HSP phenotypes. Although *ALS2* is classically linked to juvenile ALS, its role in infantile-onset ascending hereditary spastic paralysis (IAHSP) with bulbar involvement [87, 88] underscores a recognized clinical continuum with HSP, as observed in proband HSP141, who demonstrated a phenotype similar to previously reported IAHSP patients (Supplementary file S1). Similarly, *MTPAP* variants typically cause spastic ataxia type 4 (SPAX4), illustrating clinical overlaps between HSP and hereditary ataxia. All reported SPAX4 patients presented with spasticity along with ataxia, optic atrophy, and behavioral problems [89], as observed in our previously reported family, HSP159 [52].

#### Subgroup 3 (alternative diagnoses; *MFN2*, *KIF1B*, *CACNA1A*, *SPTBN2*, *PRDM8*, *SPEG*, and *MYH2*)

It includes genes associated with neurological disorders that may clinically mimic HSP but are more likely to represent an alternative diagnosis. Although these disorders share some clinical features with HSP, molecular findings demonstrate distinct genetic etiologies. Thus, this subgroup highlights the diagnostic value of molecular analysis in distinguishing HSP from alternative conditions and suggests that these patients could be reclassified.

For example, variants in *MFN2* and *KIF1B*, well-established causes of Charcot-Marie-Tooth disease [90, 91], were identified in three probands initially suspected to have c-HSP, in whom neuropathy was one of the c-HSP features. In family HSP155, a concurrent variant in *ERLIN2*/SPG18 further complicated interpretation of the neuropathy phenotype, because *ERLIN2* variants have also been associated with neuropathy [45]. Similarly, variants in *CACNA1A* and *SPTBN2*, which are known causes of hereditary ataxias [92–94], were also identified in families HSP125 and HSP219, both clinically suspected to have c-HSP accompanied by ataxia. Additionally, variants in *SPEG* and *MYH2*, which are classically associated with hereditary myopathies [95, 96], were detected in two probands who were clinically suspected of having HSP. Finally, *PRDM8* variants typically cause progressive myoclonic epilepsy type 10 (EPM10) accompanied by spastic tetraplegia [97]. In this study, two siblings from family HSP100 were referred to as suspected c-HSP patients, but the identification of a variant in *PRDM8*, suggested that the patients may be affected with early-onset Lafora disease (a severe form of progressive myoclonus epilepsy) [48].

### Group C: Emerging novel candidate genes with potential involvement in HSP

In our study, four novel candidate genes, including *SEMA3A*, *NMNAT1*, *KCNJ14*, and *EMP3* (Supplementary file S4), were detected in three HSP probands. These genes (Table 1) have not been associated with HSP, previously. It is imperative to acknowledge that our findings do not assume these novel genes are disease-causing, but rather identify them as suitable candidates based on various factors, including neuronal expression, relevant function, co-segregation analysis, and in silico prediction tools. Further functional studies and identification of additional cases are crucial to establishing these genes as disease-causing.

#### *SEMA3A* (semaphorin 3A)

A likely pathogenic heterozygous variant in the *SEMA3A* gene (c.1860+1 G > A), was identified in family HSP119 as the only candidate variant. The *de novo* nature of this variant was confirmed by co-segregation analysis. *SEMA3A* shows strong DECIPHER haploinsufficiency (HI) metrics (HI = 1.46), indicating a high probability of HI, supporting a dominant loss-of-function (LOF) mechanism. In contrast, population-based intolerance metrics show only a moderate probability (LOFtool = 0.447, RVIS percentile = 73.68). *SEMA3A* plays an essential role in the development of the nervous system, neural connections, and axonal guidance [98–102]. Semaphorin 3A is a secreted protein involved in growth cone collapse and apical dendrite growth [99–101]. Ferretti et al. demonstrated the role of semaphorin 3A in the maintenance of neuronal polarity in a recent in vitro study on human progenitor neurons [98]. Szczurkowska et al. showed that semaphorin 3A promotes apical dendrite growth and helps establish bipolar morphology in developing pyramidal neurons. Although *SEMA3A* has not yet been directly studied in UMN degeneration, its identification in our patient may indicate a potential role in axonogenesis disruption, a key feature in HSP pathogenesis.

#### *NMNAT1*

A homozygous pathogenic variant has only been reported in our previously described HSP126 proband, and was shown to co-segregate with the disease [54]. A dominant LOF mechanism is unlikely (HI = 62.8). Intolerance metrics indicate moderate to low tolerance to LOF (LOFtool = 0.3, RVIS percentile = 64.32). Recent evidence suggests that disruptions in NAD synthesis, where *NMNAT1* plays a key role, may contribute to neurodegeneration by impairing NAD’s axonal protective functions [103].

In proband HSP110, two homozygous VUS variants were identified in *KCNJ14* and *EMP3*, both co-segregating with the disease. More investigation is needed to confirm which gene as a possible disease-causing candidate.

#### *KCNJ14* (kir2.4)

There is some evidence suggesting its potential role in the pathogenesis of the disease observed in our proband. Although *KCNJ14* is not predicted to be HI (HI = 44.3), it shows strong intolerance to homozygous variants, as indicated by a low LOFtool score (0.0839). It is involved in cardiac conduction, synaptic transmission, and immune regulation [104]. In situ hybridization in the rat brain revealed that Kir2.4 has motor neuron-specific expression as well as specific motor nuclei [105], some of which establish synapses with the motor cortex of the brain, and finally innervate skeletal muscles of the pharynx, larynx, and shoulder movements [106]. Notably, severe dysarthria, dysphagia, and upper-limb involvements observed in our proband, may point to disruption of the motor nuclei pathway mentioned above. Also, pathogenic variants in various *KCNJ* genes, including *KCNJ10*, *KCNJ11*, and *KCNJ6* [107–109] are already known to cause diverse neurological disorders. Additionally, other potassium channel proteins, like KCNA2, have been reported as a cause of HSP and ataxia [110].

#### *EMP3*

*EMP3* expression was demonstrated in the developing neuronal populations belonging to the central (CNS) and peripheral (PNS) nervous systems, and Schwann cells in mouse models. Specifically, it may have a role in myelination in developing PNS by mediating axon-Schwann cell interactions [111]. Notably, impairment in myelination-related functions has been reported for some HSP subtypes, including SPG1/*L1CAM*, SPG2/*PLP1*, and SPG75/*MAG* [112]. Furthermore, a dominant LOF mechanism is unlikely for *EMP3* (HI = 40.05). However, it is compatible with an AR model (pREC = 0.154). Although population-based intolerance metrics indicate moderate tolerance to LOF (LOFtool = 0.544, RVIS percentile = 61.28), these metrics do not preclude pathogenicity due to complete biallelic loss. Overall, these results support a possible AR loss-of-function mechanism, compatible with our homozygous variant.

Based on identified genes, HSP pathogenesis involves multiple cellular pathways, including axon guidance, mitochondrial function, cytoskeletal organization, lipid metabolism, endoplasmic reticulum–Golgi transport, myelination, membrane dynamics, and nucleotide metabolism [6]. Enrichment analysis of the 21 HSP-related genes identified in this study, by the Enrichr software (https://maayanlab.cloud/Enrichr/), showed strong involvement in neurodegeneration, metabolism, and membrane trafficking pathways. Notably, the 16 newly identified candidate genes showed similar pathway enrichment, with additional novel pathways such as vitamin and cofactor metabolism, suggesting previously unrecognized mechanisms in HSP (Supplementary file S5). Understanding these pathways may help guide future therapeutic strategies, such as vitamin supplementation.

**Fig. 10:**
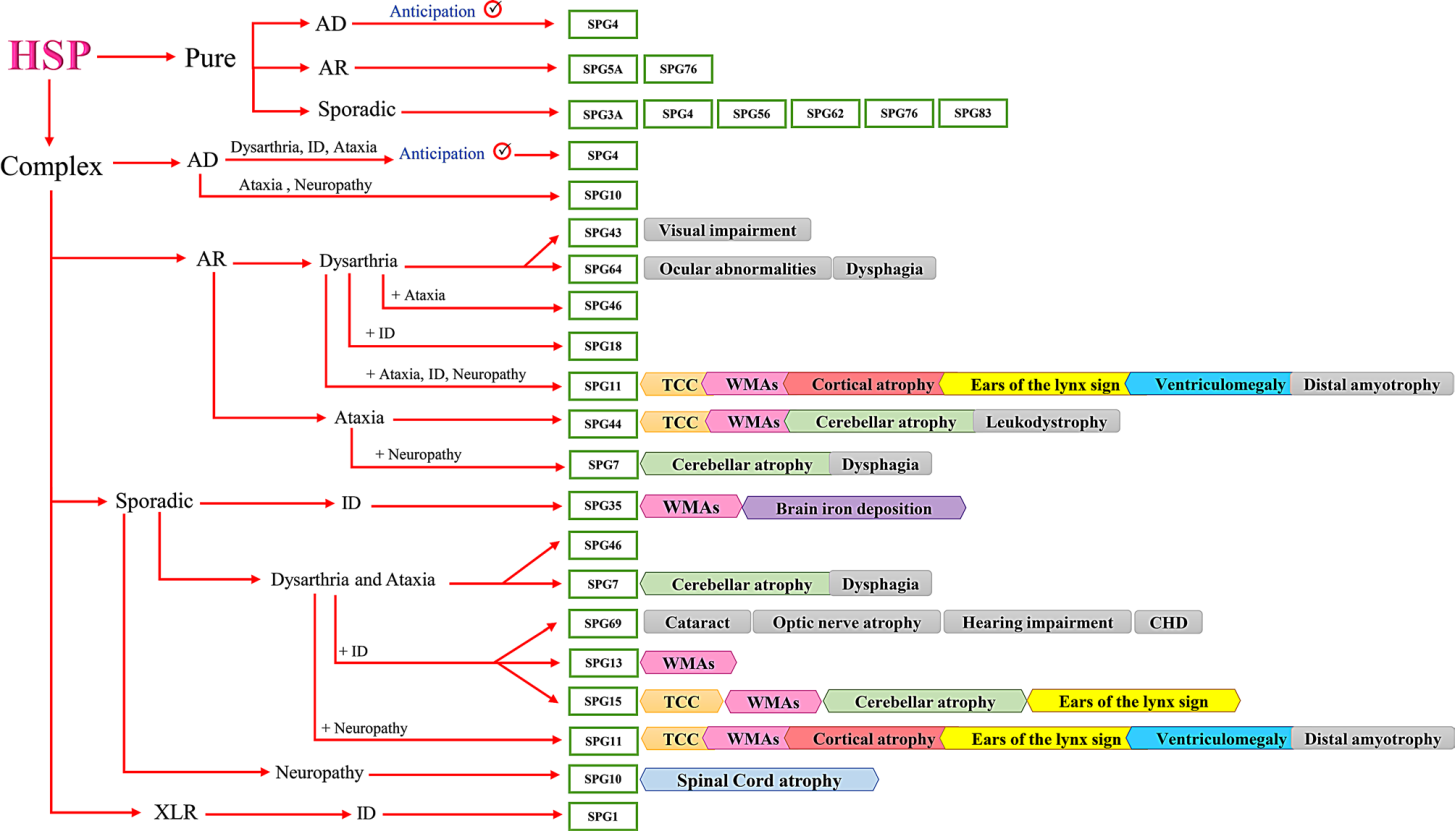
Diagnostic flowchart for Iranian HSP patients. XLR: X-linked recessive, AD: autosomal dominant, AR: autosomal recessive, TCC: thin corpus callosum, WMAs: white matter abnormalities, ID: intellectual disability, CHD: congenital heart disease

Given the lack of a standardized genetic testing protocol for HSP in Iran and the disease’s heterogeneity, we propose a comprehensive gene-based diagnostic flowchart to improve subtype identification in Iranian patients (Fig. 10).

## Conclusion

The primary diagnostic yield in this study was 70.8%, with an HSP-specific rate of 57.2%, underscoring the significant genetic heterogeneity in our cohort. SPG11 was the most common subtype, likely reflecting the high rate of consanguinity in our population. Additionally, we identified 14 probands harboring variants in genes previously linked to other neuromuscular/neurodegenerative diseases. Some of these genes can be further considered for potential inclusion in the HSP-causing gene spectrum. Such overlaps also underscore the shared biological pathways across these disorders. Overall, our findings expand the mutational and phenotypic spectrum of HSP, reinforcing the importance of genetic analysis methods for diagnosis. We also proposed four putative novel genes in three probands requiring further functional studies to validate the potential role of these novel candidate genes in HSP. Finally, 22 probands remained without a genetic diagnosis, indicating that there might be some HSP-causing genes that are still unknown, or that causative variants are hidden outside of the exome coverage.

## Limitations

This study has a few limitations that should be acknowledged. First, not all family members were available for comprehensive clinical and paraclinical evaluations, which may have limited the discovery of additional findings. Second, EMG/NCS and MRI data were not available for all patients, which may have led to an underestimation of the true frequency of EMG/NCS abnormalities, such as peripheral neuropathy, as well as MRI abnormalities in complicated HSP cases. Third, although we identified four novel candidate genes, functional validation studies were beyond the scope of this study and will be essential to confirm their pathogenicity. Fourth, although 103 families were included in the study, this number certainly does not fully represent the disease status in the general population, and evaluation of additional cases is needed. Fifth, WES does not capture all genomic regions, where the pathogenic variants may reside. In addition, WES is not particularly useful in detecting other genetic findings, such as repeat expansions or complex structural variants, and the use of short-read whole genome sequencing or long-read sequencing approaches may be more informative. Given these limitations, and considering that the disease inheritance pattern may deviate from a conventional monogenic model towards oligogenic or polygenic patterns, 22 families in our cohort remained genetically undiagnosed.

## Electronic supplementary material

Below is the link to the electronic supplementary material.

Supplementary Material 1. Supplementary file S1: Clinical and demographic information of 103 unrelated HSP probands

Supplementary Material 2. Supplementary file S2: Electromyography/nerve conduction study findings of all available HSP probands

Supplementary Material 3. Supplementary file S3: Brain magnetic resonance imaging (MRI) findings of all 103 HSP probands

Supplementary Material 4. Supplementary file S4: Genetic characteristics of all 103 HSP probands

Supplementary Material 5. Supplementary file S5. The results of incorporating the 21 HSP genes and 16 putative novel genes, identified in this study into the Enrichr software (https://maayanlab.cloud/Enrichr/) (their pathways in KEGG, Reactome, and GO)

## Data Availability

The datasets generated during and/or analyzed during the current study are available from the corresponding author upon reasonable request.
